# Effect of 30% hypertonic saline irrigation on serum electrolytes in pulmonary hydatid cyst surgery: A prospective cohort study

**DOI:** 10.1097/MD.0000000000045945

**Published:** 2025-11-07

**Authors:** Fares Abboud, Sultaneh Haddad, Mohamad Shbat, Hussain Chaban

**Affiliations:** a Faculty of Medicine, Damascus University, Damascus, Syrian Arab Republic; b Department of Pediatrics, Children’s Hospital, Damascus, Syrian Arab Republic; c Department of Thoracic Surgery, National University Hospital, Damascus, Syrian Arab Republic.

**Keywords:** echinococcosis, hypertonic saline, pulmonary hydatid cyst, serum electrolytes, surgical treatment

## Abstract

Pulmonary hydatid cysts, caused by *Echinococcus granulosus*, are treated surgically, often with intraoperative protoscolicides like hypertonic saline to prevent recurrence. The safety of 30% hypertonic saline, particularly its effect on serum electrolytes, remains underexplored in pulmonary surgery. This study evaluates the impact of 30% hypertonic saline wash on serum electrolyte levels and associated complications in pulmonary hydatid cyst surgery. A prospective cohort study was conducted at Al-Mouasat Hospital, Damascus, Syria, from June 2023 to December 2024. Twenty-two adults with pulmonary hydatid cysts undergoing surgical resection were included. Fourteen patients received 30% hypertonic saline wash, while 10 did not. Serum sodium levels were measured before, during, and 2 to 4 hours post-surgery. Data were analyzed using repeated measures analysis of variance. The cohort included 14 males (63.6%) and 8 females (36.4%). Cyst locations were 7 right lung, 7 left lung, 2 bilateral, 4 hepatopulmonary, 1 hepatic, and 1 mediastinal. No significant changes in sodium levels were observed (*F*(2,42) = 0.83, *P* = .44), with means of 140.00 (standard deviation [SD] 3.77) mmol/L presurgery, 140.82 (SD 3.29) during, and 140.73 (SD 3.43) post-surgery. No hypernatremia or related complications occurred. The use of 30% hypertonic saline wash in pulmonary hydatid cyst surgery does not significantly affect serum sodium levels and appears safe. Further studies are needed to confirm efficacy.

## 1. Introduction

Echinococcosis, also known as hydatid disease, is a zoonotic disease mainly caused by the larval stage of the parasite *Echinococcus granuloses* due to the ingestion of vegetables, fruits, and drinking water contaminated with parasitic eggs originating from the feces of infected creatures. The parasitic larvae breach the intestinal wall to reach the systemic circulation where it can attach to organs. Once attached, the larvae develop into cysts that cause varying problems based on their site and size.^[1]^ A study showed that 37% of 763 cases of infection were small uncomplicated cysts that are asymptomatic.^[2]^ Clinical symptoms occur when the cysts grow large enough to exert mechanical effects on structures or when the cyst ruptures which causes sudden onset of chest pain, cough, fever, and hemoptysis.^[3]^

No recent studies assess the prevalence of human echinococcosis in Syria, but epidemiological evidence in Syria showed a prevalence of *E granulosus* infection ranging between 9% and 15% in dogs and between 5% and 17% in livestock, which poses significant risks for the population, especially in the country’s decreasing health safety standards.^[4]^

The most common organ involved in adults is the liver, at 60%, followed by the lung, at 20% to 30%. The right lung is more commonly involved than the left lung.^[3,5]^ Concomitant hepatic cysts with lung cysts are more common in adults, while in children lung involvement is more common in addition to isolated lung cysts being more common.^[6]^ Cysts in the lungs are usually solitary and unilateral.^[7]^

While treatment options vary, the most appropriate for pulmonary hydatid cyst disease is surgery, due to its association with low morbidity and mortality rates. The treatment consists of cyst membrane extraction without manipulating the pericyst and closure of the small airways. Radical pulmonary resection should be reserved for more complicated forms of the disease.^[7,8]^

There are a handful of agents used intraoperatively for protoscolice killing. No agent showed superior efficacy or safety over the other. The lethal action of the agent in virto may be impeded in vivo by the instability of the substance used, for example hydrogen peroxide, or by unpredictable dilution by hydatid fluid, and difficulties in penetrating the cysts. Currently, the following protoscolicides have shown sufficient efficacy with a relatively low risk of toxicity: 70% to 95% ethanol, 15% to 30% hypertonic saline, or 0.5% cetrimide solution. According to expert consensus, 20% hypertonic saline is recommended.^[8–10]^

In this cohort study, we assessed the usage of 30% hypertonic saline and whether it affects serum electrolytes in a meaningful manner or not.

### 1.1. Problems of the study

The rarity of pulmonary hydatid cysts in Syria limited the sample size to 22 patients. Some eligible patients declined participation in data collection, and others were excluded due to incomplete laboratory data, particularly missing 3-hour postsurgical labs. The single-center design at Al-Mouasat Hospital may limit generalizability. The amount of hypertonic irrigation wash was not standardized and was calculated on a case by case basis depending on the irrigation area, while this is a problem for replicability, it does mimic a real-life scenario of how it is handeled. Variability in surgical techniques and potential dilution of hypertonic saline by hydatid fluid could affect outcomes.^[11]^ This study addresses the gap in understanding the safety of 30% hypertonic saline in pulmonary surgery.

### 1.2. Objectives of the study

#### 1.2.1. General objective

To evaluate the effect of 30% hypertonic saline wash on serum electrolyte levels and associated complications in pulmonary hydatid cyst surgery.

#### 1.2.2. Specific objectives of the study

1. To measure changes in serum sodium levels before, during, and 2 to 4 hours after surgery with 30% hypertonic saline wash.
2. To assess the incidence of hypernatremia and other complications associated with 30% hypertonic saline.
3. To compare electrolyte profiles between patients receiving 30% hypertonic saline wash and those not receiving it.

#### 1.2.3. Previous studies

Hypertonic saline (15–30%) is a widely used protoscolicide in hydatid cyst surgery due to its efficacy and low toxicity.^[8,9]^ Rai et al reported effective use of hypertonic saline in pulmonary hydatid surgery without significant complications, though electrolyte changes were not detailed.^[10]^ Tappeh et al demonstrated that 30% hypertonic saline effectively kills protoscoleces in vitro, but in vivo efficacy may be reduced by dilution or poor cyst penetration.^[11]^ In hepatic cyst surgery, Zeng et al and Kuzmanovska et al reported hypernatremia risks with hypertonic saline, necessitating careful monitoring.^[12,13]^ No studies have specifically evaluated the impact of 30% hypertonic saline on serum electrolytes in pulmonary hydatid cyst surgery, highlighting the need for this investigation.

## 2. Materials and methods

### 2.1. Materials

Thirty percent hypertonic saline was used for intraoperative irrigation of the operative surface of opened cavity for protoscolice killing after injecting and excising the cyst.

- Population of the study: The study included 22 adults (males and females) with pulmonary hydatid cysts undergoing surgical resection at Al-Mouasat Hospital, Damascus, Syria.
- Design of study: Prospective cohort study conducted from June 2023 to December 2024.
- Area of study: This study was conducted in Al-Mouasat Hospital, Damascus, Syria.
- Sampling: A sample of 22 adults with varying age, weight, height, and gender were included. Twelve patients received 30% hypertonic saline irrigation, and 10 did not.

### 2.2. Methods

- Techniques: Cysts were injected with 20 to 30 mL of hypertonic saline then resected, and cavities in the intervention group were irrigated with a per-case amount of 30% hypertonic saline that is enough for adequate coverage of the rescection area. Serum sodium levels were measured before, during, and at 2, 3, and 4 hours post-surgery for both study groups.
- Data collection: Data were recorded on patient-specific forms, including name, age, cyst location, surgical approach, and laboratory results (sodium levels).
- Data analysis method: Repeated measures analysis of variance was used to compare sodium levels across time points. Statistical significance was set at *P* < .05.

### 2.3. Ethical considerations

The study was approved by the Research Licensing Committee at Damascus University. Permission was obtained from Al-Mouasat Hospital’s administrator. Verbal consent was obtained from participants, ensuring privacy and ethical standards. No identifying information was published.

## 3. Results

The cohort consisted of 14 males (63.6%) and 8 females (36.4%). Cyst locations included 7 right lung, 7 left lung, 2 bilateral, 4 hepatopulmonary, 1 hepatic, and 1 mediastinal. 12 patients received 30% hypertonic saline wash, and 10 did not.

**Figure 1.**
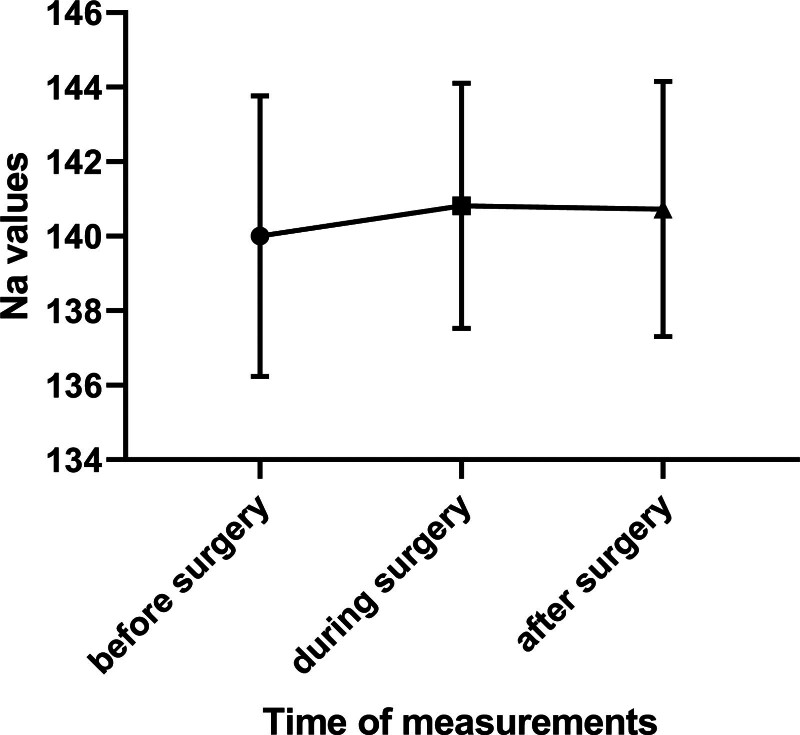
Average serum sodium levels before, during, and after surgery.

Repeated measures analysis of variance revealed no significant difference in sodium levels (*F*(2,42) = 0.83, *P* = .44). Mean sodium levels (mmol/L) were 140.00 (standard deviation [SD] 3.77) presurgery, 140.82 (SD 3.29) during, and 140.73 (SD 3.43) post-surgery. Figure 1 shows the average sodium levels with compression of the 2, 3, and 4 hours after surgery measurements into 1 column. No hypernatremia or other complications were observed.

## 4. Discussion

This prospective cohort study evaluated the safety of 30% hypertonic saline irrigation during hydatid cyst surgery, focusing on its impact on serum sodium levels. The results demonstrated no significant changes in sodium levels across the measured time points (F(2,42) = 0.83, *P* = .44), with mean sodium levels remaining stable at 140.00 (SD 3.77) mmol/L presurgery, 140.82 (SD 3.29) during surgery, and 140.73 (SD 3.43) post-surgery. Additionally, no instances of hypernatremia or other complications were observed, suggesting that 30% hypertonic saline is a safe protoscolicide in this surgical context.

The absence of significant electrolyte changes is a notable finding, particularly when compared to prior studies. Rai et al reported no major complications with hypertonic saline irrigation in pulmonary hydatid cyst surgery, although their study did not specifically measure serum electrolyte levels.^[10]^ This consistency with our findings strengthens the evidence for the safety of hypertonic saline in pulmonary surgery. However, studies on hepatic hydatid cyst surgery have reported hypernatremia as a potential risk associated with hypertonic saline use.^[12,13]^ For example, Zeng et al documented cases of hypernatremia in hepatic cyst surgery, attributing it to systemic absorption of hypertonic saline through the highly vascular liver tissue.^[12]^ Kuzmanovska et al further highlighted neurologic complications due to hypernatremia in hepatic surgery, emphasizing the need for careful monitoring.^[13]^ The contrast between these hepatic studies and our pulmonary findings may be explained by differences in tissue vascularity. Pulmonary tissue, being less vascular than hepatic tissue, may limit systemic absorption of hypertonic saline, thereby reducing the risk of electrolyte imbalances.^[1]^ This hypothesis is supported by the anatomical characteristics of pulmonary cysts, which are often solitary and encapsulated, potentially minimizing leakage into the bloodstream.^[5,7]^ While our sample was aimed for diversity of cyst location, only 5 out of 22 patients had hepatic cysts, and out of these 5 patients, 3 were selected for hypertonic fluid treatment. Reviewing their lab values showed no deviation from the results.

The safety profile observed in this study also aligns with the in vitro efficacy of 30% hypertonic saline as a protoscolicide. Tappeh et al demonstrated that 30% hypertonic saline effectively kills protoscoleces, although it is in vivo efficacy may be reduced by dilution with hydatid fluid or poor penetration into the cyst wall.^[11]^ Our study did not assess protoscolicidal efficacy directly, as the focus was on safety and electrolyte changes. However, the absence of complications suggests that 30% hypertonic saline can be applied without adverse effects, even if its effectiveness in preventing recurrence requires further investigation. The World Health Organization guidelines and expert consensus recommend 20% hypertonic saline as the standard due to its balance of efficacy and safety.^[8,9]^ Our findings suggest that 30% hypertonic saline may be a viable alternative, particularly in settings where higher concentrations are preferred to ensure protoscolicidal activity, but this requires confirmation through studies directly measuring recurrence rates.

Several factors may have contributed to the stable electrolyte levels observed. First, the volume of hypertonic saline used during irrigation was likely controlled by surgeons to minimize systemic exposure, though exact volumes were not standardized in this study. Second, the surgical technique, which involved careful resection of the cyst membrane without manipulating the pericyst, may have reduced the risk of saline leakage into surrounding tissues.^[7]^ Third, the timing of electrolyte measurements (before, during, and 2–4 hours post-surgery) was sufficient to detect acute changes, suggesting that any potential absorption of hypertonic saline was minimal or rapidly corrected by physiological mechanisms. These factors underscore the importance of standardized protocols in surgical practice to ensure consistent outcomes.

Despite these promising results, the study has several limitations that warrant consideration. The small sample size (n = 22) was constrained by the rarity of pulmonary hydatid cysts in Syria even though animals are infected more than the global average,^[4]^ patient refusals to participate in data collection, and exclusions due to incomplete laboratory data, due to delays from insufficient staffing. This limited statistical power, potentially masking subtle electrolyte changes that might be detected in a larger cohort. The single-center design at Al-Mouasat Hospital may not reflect variations in surgical practices or patient demographics in other settings, limiting generalizability. Additionally, the lack of a comparator group receiving a different protoscolicide (e.g., 20% hypertonic saline or ethanol) prevents direct assessment of relative safety and efficacy. Variability in surgical techniques, such as differences in irrigation volume or duration, could also introduce confounding factors, though this was not quantified in the study. Finally, the study did not evaluate long-term outcomes, such as cyst recurrence rates, which are critical for assessing the overall effectiveness of 30% hypertonic saline.

The findings have important clinical implications, particularly in resource-limited settings like Syria, where echinococcosis remains a public health concern due to high prevalence in dogs and livestock.^[4]^ The safety of 30% hypertonic saline supports its use in pulmonary hydatid cyst surgery, especially in hospitals with limited access to alternative protoscolicides like cetrimide or high-concentration ethanol. Routine electrolyte monitoring, as performed in this study, can further ensure patient safety. However, clinicians must remain vigilant for potential dilution effects, as noted by Tappeh et al, which could reduce protoscolicidal efficacy.^[11]^ Standardizing the volume and application method of hypertonic saline could enhance its reliability in clinical practice.

Future research should address the limitations of this study to advance the evidence base. Multicenter studies with larger sample sizes are needed to confirm the safety and efficacy of 30% hypertonic saline across diverse populations and surgical settings. Comparative trials evaluating 30% versus 20% hypertonic saline or other protoscolicides (e.g., 70–95% ethanol, 0.5% cetrimide) would clarify the optimal agent and concentration. Additionally, studies measuring protoscolicidal efficacy, such as recurrence rates or intraoperative protoscolex viability, are essential to complement safety data. Investigating methods to mitigate dilution by hydatid fluid, such as improved irrigation techniques or adjuvants, could enhance in vivo effectiveness. Finally, exploring patient-specific factors (e.g., cyst size, location, or comorbidities) that influence outcomes could guide personalized treatment protocols.

## 5. Conclusion

This study provides valuable evidence that 30% hypertonic saline wash is safe in pulmonary hydatid cyst surgery, with no significant impact on serum sodium levels. While these findings are encouraging, they highlight the need for further research to establish optimal protoscolicidal strategies and ensure their effectiveness in preventing recurrence, particularly in endemic regions like Syria.

## 6. Recommendations

- Clinicians may use 30% hypertonic saline as a safe protoscolicide in pulmonary hydatid cyst surgery, with routine electrolyte monitoring.
- Future studies should investigate larger, multicenter cohorts to enhance generalizability.
- Comparative studies of 30% versus 20% hypertonic saline or other protoscolicides are needed to determine optimal safety and efficacy.
- Research should explore methods to mitigate dilution of hypertonic saline by hydatid fluid.

## Author contributions

**Conceptualization:** Fares Abboud, Sultaneh Haddad.

**Data curation:** Fares Abboud, Mohamad Shbat.

**Formal analysis:** Fares Abboud.

**Investigation:** Fares Abboud.

**Methodology:** Fares Abboud.

**Project administration:** Sultaneh Haddad, Mohamad Shbat.

**Resources:** Mohamad Shbat.

**Supervision:** Hussain Chaban.

**Validation:** Fares Abboud.

**Visualization:** Fares Abboud.

**Writing – original draft:** Fares Abboud, Sultaneh Haddad.

**Writing – review & editing:** Fares Abboud, Sultaneh Haddad, Mohamad Shbat, Hussain Chaban.
